# Origin and evolution of a placental-specific microRNA family in the human genome

**DOI:** 10.1186/1471-2148-10-346

**Published:** 2010-11-10

**Authors:** Zhidong Yuan, Xiao Sun, Dongke Jiang, Yan Ding, Zhiyuan Lu, Lejun Gong, Hongde Liu, Jianming Xie

**Affiliations:** 1 State Key Laboratory of Bioelectronics, School of Biological Science and Medical Engineering, Southeast University, Nanjing, China; 2 School of Life Science, Hunan University of Science and Technology, Xiangtan, China

## Abstract

**Background:**

MicroRNAs (miRNAs) are a class of short regulatory RNAs encoded in the genome of DNA viruses, some single cell organisms, plants and animals. With the rapid development of technology, more and more miRNAs are being discovered. However, the origin and evolution of most miRNAs remain obscure. Here we report the origin and evolution dynamics of a human miRNA family.

**Results:**

We have shown that all members of the miR-1302 family are derived from MER53 elements. Although the conservation scores of the MER53-derived pre-miRNA sequences are low, we have identified 36 potential paralogs of MER53-derived miR-1302 genes in the human genome and 58 potential orthologs of the human miR-1302 family in placental mammals. We suggest that in placental species, this miRNA family has evolved following the birth-and-death model of evolution. Three possible mechanisms that can mediate miRNA duplication in evolutionary history have been proposed: the transposition of the MER53 element, segmental duplications and Alu-mediated recombination. Finally, we have found that the target genes of miR-1302 are over-represented in transportation, localization, and system development processes and in the positive regulation of cellular processes. Many of them are predicted to function in binding and transcription regulation.

**Conclusions:**

The members of miR-1302 family that are derived from MER53 elements are placental-specific miRNAs. They emerged at the early stage of the recent 180 million years since eutherian mammals diverged from marsupials. Under the birth-and-death model, the miR-1302 genes have experienced a complex expansion with some members evolving by segmental duplications and some by Alu-mediated recombination events.

## Background

MiRNAs are endogenously expressed, single-stranded RNAs ~22 nucleotides (nt) in length [1]. In animals, miRNAs are transcribed as long primary miRNA (pri-miRNA) sequences that are processed in the nucleus to give precursor sequences of miRNA (pre-miRNAs). The pre-miRNA sequences are exported to the cytoplasm where they are cleaved to produce mature miRNAs. The miRNAs are then incorporated into RNA-induced silencing complexes where they function either to inhibit translation or to mediate the degradation of their target mRNAs commonly by binding to complementary regions in the 3' untranslated regions (UTRs) [2-4]. MiRNAs play a pivotal role in many cellular functions by regulating normal developmental and physiological processes [5-7], and are involved in disease development [8,9].

Repetitive elements (repeats) include tandem repeats and interspersed repeats (DNA transposons and retrotransposons). Interspersed repeats are responsible for gene (or exon) shuffling and duplication [10,11] as well as for regulatory changes [12,13]. Gene (or exon) shuffling and duplication leads to the de novo creation of protein domains [12,14] or new protein sequences [15,16]. Recently, many miRNAs derived from repetitive elements have been identified in mammals and plants [17-21]. Some transposable elements become integrated in multiple loci in the genome and evolve into different members of a miRNA family. An example of this is the hsa-mir-548 family, the members of which are derived from Made1 transposable elements [19]. Some transposable elements surrounding miRNAs have also been found to facilitate the expansion of miRNA clusters [18,22]. As the evolution of many miRNAs remains obscure, the analysis of miRNAs derived from repetitive elements may facilitate the understanding of the evolution of miRNAs.

Here we report our study of the miR-1302 gene family that has been experimentally verified in the human genome [23]. In miRBase (Release 16.0, Sept 2010), this family has 11 members distributed in the human genome. Members of this family have recently been identified using computational methods in the chimpanzee and horse genomes [24,25]. We have found that all members of this family are derived from one transposon, the MER53 element [26]. The MER53 element is a type of DNA transposable element with a 193-bp (base pair) consensus sequence that exists in eutherian species. MER53 elements are characterized by the presence of terminal inverted repeats and TA target site duplications that can form palindromic structures [26]. If integrated into the genome and transcribed, they may be processed into miRNAs by the miRNA processing machine. In this paper, we focus on MER53-derived miRNAs in the human genome. First, we identified MER53-derived miRNAs in known miRNAs and scanned the human genome for their paralogs. Next, the phylogenetic distribution and evolution dynamics of the miR-1302 family were analyzed. Finally, we investigated the functions of the predicted target genes and analyzed the over-representation of Gene Ontology terms and KEGG pathways for this miRNA family.

## Results

### Conservation Evaluation of MER53-derived miRNAs in the Human Genome

**Figure 1:**
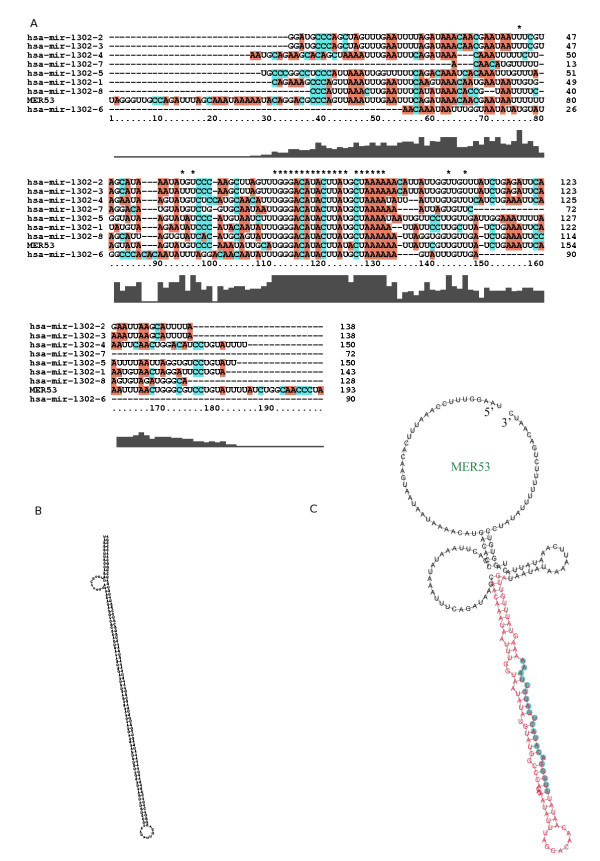
**Multiple alignment of MER53-derived miRNAs from miRBase v13.0 and the secondary structures of MER53 elements**. (A) The multiple alignment of MER53 and the eight identified MER53-derived sequences of hsa-mir-1302. (B) The secondary structure of an MER53 element showing its palindromic stem-loop structure. The MER53 element was retrieved from the RepeatMasker Database. (C) An example of the secondary structure of an MER53 element that harbors the precursor and mature sequence of hsa-mir-1302-6. The hairpin structure of the precursor of hsa-mir-1302-6 is shown in red and the segment corresponding to the mature miRNA is highlighted in light blue.

By comparing the genomic coordinates of human miRNAs from miRBase v13.0 [27] with the locations of repeats annotated by RepeatMasker [28] in the human genome, we found 11 MER53-derived miRNA genes that can encode hsa-mir-1302-1, hsa-mir-1302-2 (four copies on different chromosomes), hsa-mir-1302-3, hsa-mir-1302-4, hsa-mir-1302-5, hsa-mir-1302-6, hsa-mir-1302-7 and hsa-mir-1302-8 (Figure 1, Table 1 and Figure S1 in Additional File 1). In miRBase v13.0, there were eight members of the hsa-mir-1302 family. Four identical copies of hsa-mir-1302-2 were identified on chromosomes 1, 9, 15 and 19 (Table 1 and Figure S1 in Additional File 1). In the later updates of miRBase, hsa-mir-1302-2(chr9), hsa-mir-1302-2(chr15) and hsa-mir-1302-2(chr19) were renamed as hsa-mir-1302-9 (chr9), hsa-mir-1302-10 (chr15) and hsa-mir-1302-11 (chr19) respectively. The IDs of the other members of the miR-1302 family are unchanged. Except for hsa-mir-1302-5, the coverage densities of MER53 sequences to these pre-miRNAs are 100% (Table 1). It is interesting that all 11 of the MER53-derived miRNA genes that we identified, developed into one mature miRNA, hsa-miR-1302 (5'UUGGGACAUACUUAUGCUAAA3').

**Table 1:** Percentage and Conservation Scores for MER53-derived hsa-mir-1302 Members Embedded in Repeats

miRNA Gene		Repeat		Percent	Score
		
Coordinate	miRNA	Coordinate	MER53		
chr1:20229-20366(+)	miR1302-2	chr1:20206-20395(-)	MER53	100%	0.06
chr2:114057006-114057143(-)	miR1302-3	chr2:114056977-114057166(+)	MER53	100%	0.15
chr2:207842244-207842393(-)	miR1302-4	chr2:207842230-207842416(-)	MER53	100%	0.2
chr7:18133368-18133457(-)	miR1302-6	chr7:18133317-18133525(-)	MER53	100%	0.02
chr8:142865510-142865581(-)	miR1302-7	chr8:142865462-142865600(+)	MER53	100%	0.01
chr9:20144-20281(+)	miR1302-2	chr9:20121-20310(-)	MER53	100%	0.06
chr9:99165657-99165784(-)	miR1302-8	chr9:99165632-99165820(-)	MER53	100%	0.02
chr12:111617222-111617364(-)	miR1302-1	chr12:111617205-111617395(-)	MER53	100%	0.09
chr15:100318185-100318322(-)	miR1302-2	chr15:100318156-100318345(+)	MER53	100%	0.11
chr19:22973-23110(+)	miR1302-2	chr19:22950-23139(-)	MER53	100%	0.06
chr20:48664580-48664729(-)	miR1302-5	chr20:48664721-48665024(+)	AluSx1	99.33%	0
		chr20:48664577-48664719(+)	MER53		

miR1302: An abbreviation of hsa-mir-1302

Coordinate: The genomic location of the miRNAs and repeats in the hg18 genome assembly

Percent: The coverage of the MER53 element in the pre-miRNA

Score: The average conservation score of the pre-miRNA

The average phastCons conservation scores of pre-miRNA sequences have been used to determine the conservation of pre-miRNA. In our study, the average phastCons conservation scores of hsa-mir-1302 members are much lower than the thresholds used in previous studies (Table 1) [21,29].

### Potential Paralogs of the miR-1302 Family in the Human Genome

Of the 5,839 MER53 elements annotated in the hg18 genome assembly, we identified 44 MER53 elements that may encode miRNAs (Additional File 2). Eight of the 44 potential sequences overlap with experimentally verified miR-1302 precursor sequences [23] that are distributed on different chromosomes (Additional File 2). Hsa-mir-1302-5 and hsa-mir-1302-7 were not identified by our method as their multi-branched loops were filtered out by the MiPred program and hsa-mir-1302-8 was not identified because the selected region did not meet our criterion to be a pre-miRNA. Thus, we have identified 36 MER53 elements that may encode miRNAs belonging to the miR-1302 family and that have not yet been reported in the human genome.

### Phylogenetic Distribution of Orthologs of the Human miR-1302 Family

**Figure 2:**
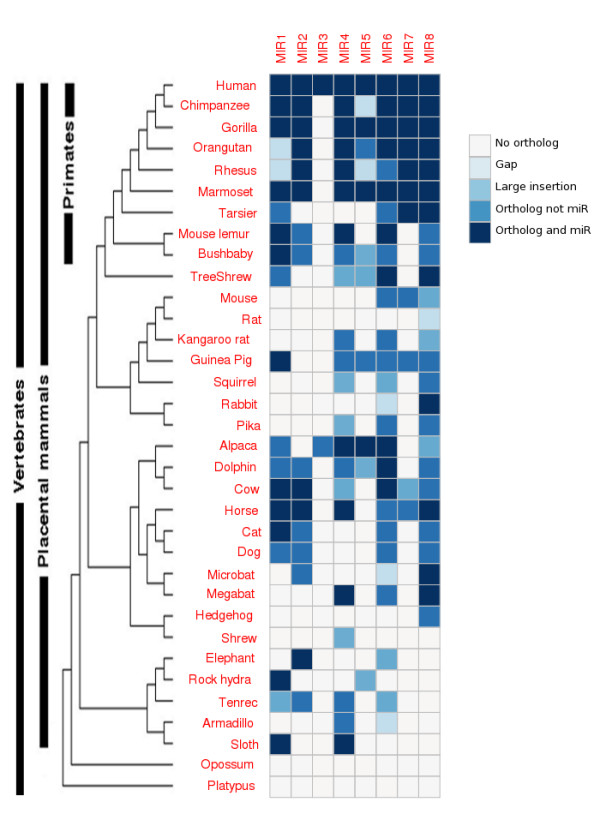
**Phylogenetic distribution of orthologs of the hsa-mir-1302 family**. The phylogenetic tree was modified from the UCSC Genome Browser. Although in human there are 4 identical copies of hsa-mir-1302-2, in all other the species listed there is only one. Abbreviations: miR, miRNA; Gap, a gap in the genome; Large insertion, an insertion of more than 100 bp, except for a 17 bp insertion in the ortholog of hsa-mir-1302-4 in Pika; Ortholog not miR, the ortholog is not a miRNA; Ortholog and miR, the ortholog has been validated as a miRNA; MIR1-MIR8, the orthologs of hsa-mir-1302-1, hsa-mir-1302-2, hsa-mir-1302-3, hsa-mir-1302-4, hsa-mir-1302-5, hsa-mir-1302-6, hsa-mir-1302-7, and hsa-mir-1302-8 respectively.

The 103 orthologs (Figure 2) of the hsa-mir-1302 family, identified as described in Materials and Methods, were only found in placental mammals, suggesting that the miR-1302 family is a placental-specific gene family. Of these, 58 orthologs from 21 species were validated as miRNA using MiPred [30] (see Additional File 3). The 21 species are chimpanzee, gorilla, orangutan, rhesus, marmoset, tarsier, mouse lemur, bushbaby, tree shrew, guinea pig, rabbit, alpaca, dolphin, cow, horse, cat, microbat, megabat, elephant, rock hyrax and sloth (Figure 2). Although there are four copies of the hsa-mir-1302-2 gene in the human genome, only one ortholog was found in the genomes of all 21 species.

MER53 elements are only found in eutherian species (placental mammals) [26]. Because eutherian mammals diverged from marsupials and monotremes 180 and 210 million years ago, respectively [31], the homologs of the hsa-mir-1302 family in placental mammals may all be derived from MER53 elements, explaining why homologs of the mir-1302 family are not found in opossum and platypus (Figure 2). From this, we can infer that MER53 elements and MER53-derived miRNA genes emerged at the early stage of the recent 180 million years since eutherian mammals diverged from marsupials.

During evolution, many miR-1302 genes have been gained and lost (Figure 2). To estimate the gain and loss of miR-1302 genes during evolution, we have used the previously reported parsimony method [32] to infer that miR-1302-1, miR-1302-2, miR-1302-4, miR-1302-5, miR-1302-6, miR-1302-7, and miR-1302-8 evolved after the MER53 elements had been inserted and fixed. For example, miR-1302-2, miR-1302-4 and miR-1302-5 gene are present in human and marmoset but absent in other species such as tarsier, suggesting that these genes were generated in the ancestor of human and marmoset and lost in tarsier (Figure 2). In addition, in the tarsier genome the orthologs of miR-1302-1 and miR-1302-6 are further diverged than other miRNA genes (Figure 2). In humans, three additional hsa-mir-1302-2 genes and one hsa-mir-1302-3 have been duplicated from the original hsa-mir-1302-2 (discussed in detail in the next section). The miRNA gene family has experienced repeated gene duplication and while some of the duplicated genes have diverged functionally others have become pseudo genes or have been deleted from the genome of these species. This pattern is clearly shown in Figure 2.

Previous workers have pointed out that if the gene members of a family evolve in a birth-and-death manner the genes will cluster by type and not by species, while under the concerted model they will cluster by species [33,34]. Except for hsa-1302-2 and hsa-1302-3 that are produced from recent segmental duplication events and cluster together, the miR-1302 genes do not show a within-species clustering pattern (Figure S2 in Additional File 1). Combining the evolutionary divergence between sequences information (Additional file 4) and the results of previous analysis, we can infer that the miR-1302 genes evolved in a birth-and-death manner.

### Segmental Duplication and Alu Repeats Mediate the Expansion of the miR-1302 Gene in the Human Genome

**Figure 3:**
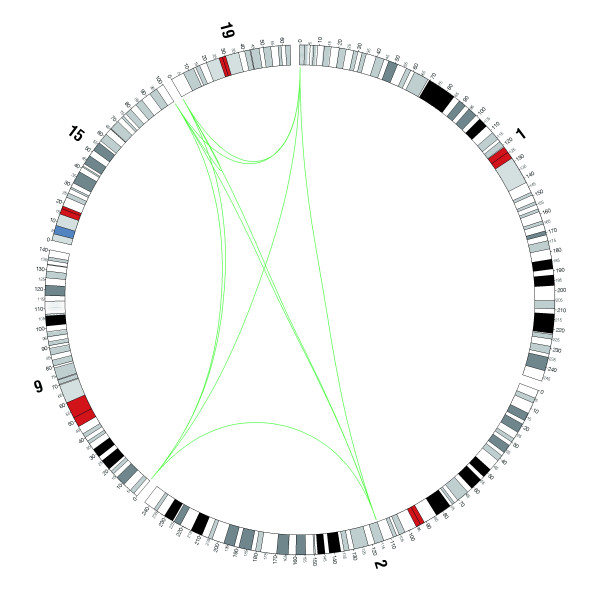
**Relationship between segmental duplication pairs in the human genome**. The relationships are shown between the 10 duplication pairs where the miR-1302 genes located on chromosomes 1, 9, 15 and 19 are linked by green lines. The four copies of hsa-mir-1302-2 are distributed on chromosomes 1, 9, 15 and 19. Hsa-mir-1302-3 is on chromosome 2. The circular ideograms of the chromosomes were drawn using the files of genome karyotypes from the UCSC Genome Browser.

It is known that some protein-coding gene families expanded by segmental duplication events [35-39]. Some of the eleven members of the hsa-mir-1302 family are remarkably similar. There are four identical copies of the hsa-mir-1302-2 gene on different chromosomes and hsa-mir-1302-2 and hsa-mir-1302-3 differ at only two nucleotide positions (see Figure S3A in Additional File 1). We therefore hypothesize that, in addition to the MER53 transposition effect, segmental duplication events may have contributed to the expansion of the hsa-mir-1302 family. If this is correct, then some members of the family should be located in regions of segmental duplications. We find that five of the miRNA genes (the four copies of hsa-mir-1302-2 and hsa-mir-1302-3) are indeed located in segmental duplications (Table 2) thus supporting this view. As shown in Table 2, there are 10 non-redundant segmental duplication pairs in the human genome, some of which overlap. The relationship between these duplications is shown in Figure 3 where each duplication pair is linked by a line. The miR-1302 gene and the neighboring genes in each duplication region are shown in Additional file 5. In each of the segmental duplication pairs, the gene members are similar and the gene order is either identical or in the opposite direction (see Additional file 5 and Figure S4 in Additional File 1). This is powerful evidence supporting our thesis that the miR-1302 gene and its neighbors evolved by segmental duplication events.

**Table 2:** Precursors of Human miR-1302 in Segmental Duplications

ID	Segmental Duplication Pair		miRNA Genes in the Duplication	
	
	Coordinate of Duplication (SD1)	Corresponding Duplication (SD2)	miRNAs in SD1	miRNAs in SD2
1	chr1:466-30596(-)	chr2:114046769-114076456(-)	miR1302-2(chr1)	miR1302-3(chr2)
2	chr1:487-76975(-)	chr15:100263880-100338121(-)	miR1302-2(chr1)	miR1302-2(chr15)
3	chr1:487-30596(+)	chr9:845-30515(+)	miR1302-2(chr1)	miR1302-2(chr9)
4	chr1:8257-76975(+)	chr19:11002-79672(+)	miR1302-2(chr1)	miR1302-2(chr19)
5	chr2:113887481-114076781(-)	chr9:413-193762(-)	miR1302-3(chr2)	miR1302-2(chr9)
6	chr2:114046769-114069083(-)	chr19:11002-33344(+)	miR1302-3(chr2)	miR1302-2(chr19)
7	chr2:114046769-114076720(+)	chr15:100307957-100338402(+)	miR1302-3(chr2)	miR1302-2(chr15)
8	chr9:437-30515(-)	chr15:100307957-100338529(-)	miR1302-2(chr9)	miR1302-2(chr15)
9	chr9:8507-30515(+)	chr19:11002-33344(+)	miR1302-2(chr9)	miR1302-2(chr19)
10	chr15:100218756-100330295(-)	chr19:11002-123445(-)	miR1302-2(chr15)	miR1302-2(chr19)

miR1302: An abbreviation of hsa-mir-1302,

miR1302-2(chr1): has-mir-1302-2 on chr1:20229-20366(+),

miR1302-2(chr9): has-mir-1302-2 on chr9:20144-20281(+),

miR1302-2(chr15): has-mir-1302-2 on chr15:100318185-100318322(-),

miR1302-2(chr19): has-mir-1302-2 on chr19:22973-23110(+),

miR1302-3(chr2): has-mir-1302-3 on chr2:114057006-114057143(-)

There is only a two-nucleotide difference between hsa-mir-1302-2 and hsa-mir-1302-3 (see Figure S3A in Additional File 1), the same as the difference in the alignment of their corresponding MER53 sequences (see Figure S3B in Additional File 1). This indicates that, owing to DNA segmental duplication, the hsa-mir-1302-2 sequence was also duplicated. When the hsa-mir-1302-2 gene underwent duplication, as part of the evolution process, it is possible that mutations occurred resulting in a new gene, hsa-mir-1302-3.

Because they have not been found in regions of segmental duplication, the other hsa-mir-1302 genes may be products of MER53 transposition events alone. To determine if they were also formed by segmental duplication events, we analyzed the 36 paralogs of the hsa-mir-1302 family that we identified in the human genome (Additional File 2). We found that, like most of the members of the hsa-mir-1302 family, none of them were located in regions of segmental duplications.

**Figure 4:**
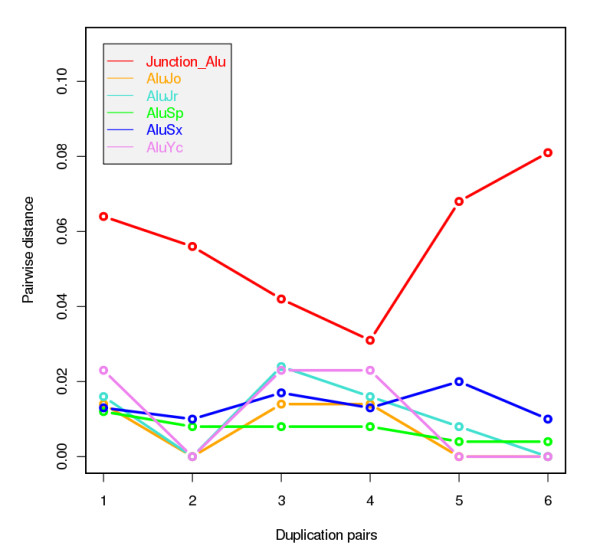
**Comparison of the sequence divergence of junction Alus and internal Alus in distinct duplication pairs**. The six duplication pairs are: 1, chr1:466-30596(-) and chr2:114046769-114076456(-); 2, chr1:487-30596(+) and chr9:845-30515(+); 3, chr2:114046769-114076720(+) and chr15:100307957-100338402(+); 4, chr2:114046769-114069083(-) and chr19:11002-33344(+); 5, chr9:437-30515(-) and chr15:100307957-100338529(-); 6, chr9:8507-30515(+) and chr19:11002-33344(+). AluJo, AluJr, AluSp, AluSx, AluYc are the internal Alus in the duplication pairs.

Alu-mediated recombination events may facilitate the expansion of segmental duplications through recombination [40] as well as mediate the expansion of miRNA genes [18,22]. Alu-mediated recombination creates mosaic Alu elements at the recombination junctions. Consequently, the junction Alu elements exhibit marked sequence divergence when compared with internal Alus [40]. In the present study, we find that for most of the segmental duplications only one of the ends (the 5' or 3' end) adjoins one or more of the Alu sequences (see Figure S4 in Additional File 1). Further, the distribution pattern of AluSp-(hsa-mir-1302-2/3)-AluJo-AluYc-AluJr-AluSx is present in all of these duplication sequences (see Figure S4 in Additional File 1). Therefore, we have used AluSp, AluJo, AluYc, AluJr and AluSx as internal Alu elements. Of the 10 duplication pairs, six pairs have Alu elements located at the junction and these were used in the present analysis. Interestingly, we find no significant sequence divergence among the internal Alu pairs (Kruskal-Wallis chi-squared = 3.0372, df = 4, p-value = 0.5516), but the sequence divergence between the junction Alu pairs and the internal Alu pairs is considerable (Kruskal-Wallis chi-squared = 16.8957, df = 5, p-value = 0.004702), indicating that the junction Alu pairs have diverged more than the internal Alu pairs (Figure 4). We therefore suggest that during the evolution of the hsa-mir-1302-2 and hsa-mir-1302-3 genes, Alu-mediated recombined events have facilitated the expansion of the segmental duplications that harbor them.

### Target Prediction and Functional Analysis

The function of hsa-miR-1302 is still unknown. We have analyzed the targets of the mature human miR-1302 in an attempt to explore its potential function. In the human genome, 1,835 target genes are predicted by both PITA [41] (17,844 predicted target genes and left 39,154 target sites if removing the duplicate coordinates, see Additional file 6) and TargetScan [42,43] (2,055 target genes with 94 conserved sites and 2,334 poorly conserved sites, see Additional file 7). We used the 1,835 target genes at the intersection between the two predicted gene sets as valid targets (Additional file 8). Ninety-one of the TargetScan predicted conserved sites (Additional file 9) and 1,744 of the TargetScan predicted poorly conserved sites were identified in the 1,835 valid target gene data set. To determine the functions and pathways that may involve hsa-miR-1302, all the valid targets of hsa-mir-1302 were annotated using WebGestalt [44] and KEGG [45]. The 1,835 target genes are widely distributed across all the chromosomes (see Figure S5A in Additional File 1) and are expressed in most tissues of the body. The highest expression levels are in the nervous system, and lowest are in the soft tissue and in the adrenal medulla (see Figure S5B in Additional File 1). In the nervous tissue, the genes are enriched in intracellular membrane-bounded organelles and in the synapse (see Figure S5C in Additional File 1). Functionally, the target genes are over-represented in transportation, localization, system development processes, and in the positive regulation of cellular processes. They may also play a role in binding and transcription regulation (see Figure S5D, Figure S5E in Additional File 1 and Additional file 10). Overall, these predicted target genes are implicated in cell proliferation and cell division, metabolism, development and in the immune response. In the pancreas some of the genes have roles in the insulin-related signal pathway and in pancreas pathology, while in the nervous system some are involved in learning, memory, and signal transduction and are implicated in neural disease development (see Figure S5 D-E in Additional File 1 and Additional file 10). The KEGG annotation indicates that some of the target genes are involved in diseases and are enriched in pathways that lead to glioma, chronic myeloid leukemia, colorectal cancer, pancreatic cancer, type II diabetes mellitus, neurodegenerative disorders such as Alzheimer's disease, and pathogenic Escherichia coli infection (Additional file 10). As miR-1302 was first identified in pluripotent human embryonic stem cells and embryoid bodies [23] and is enriched in pathways to cancer, it may influence the biological processes taking place in stem cells, in tumor cells and in the early embryo.

## Discussion

Recently, miRNAs that originate from repetitive elements have been identified in mammals and plants [17-21]. Here we report a microRNA family, the miR-1302 family, which originates from the DNA transposable element, MER53. MER53 is a medium reiteration frequency, non-autonomous DNA transposon related to the mariner family. MER53 elements can form palindromic stem-loop structures (see Figure 1B and Figure 1C) [26]. Once an MER53 element becomes inserted into the genome in the region of an active transcription and is fixed by natural selection, it may be transcribed and processed by the enzyme machine system of miRNA into miRNAs. Previous studies have shown that transposable-element-derived miRNAs are less conserved than non-transposable-element-derived miRNAs [21]. However, we find that the average conservation scores for the miR-1302 genes are very low (Table 1). Because quite a few orthologs of the precursors of human miR-1302 are found (See Additional File 3) in eutherian mammals, we suggest that the MER53 elements and MER53-derived miRNA genes may have evolved after eutherian mammals diverged from marsupials and monotremes as recently as 180 million years ago. In placental species, many miR-1302 genes have been gained and lost, indicating that they may have evolved following the birth-and-death model of evolution. It should be noted that the parsimony reconstruction of gain and loss of the miR-1302 genes is influenced by the fact that the miR-1302 genes were first identified in the human genome and the orthologs in other species were identified using computational approaches. Nevertheless, the theoretical approach is a good starting point for deducing the evolution mode for miRNA families.

MiRNAs, like protein-coding genes, form gene families and like MIR166 in plants, many of the pre-miRNAs produce either similar or identical mature miRNAs [27,46]. These pre-miRNAs are classified as one family. The following questions arise: How do miRNA genes evolve to become miRNA families? What is the evolution dynamics of these miRNA genes? And are their evolutionary patterns the same as those for protein-coding gene families? Previous studies have shown that the expansion of miRNA families usually occurs through tandem or segmental duplications [22,47-50]. In the present study, we have focused on the precursors of human miR-1302. Because genes of the miR-1302 family are found on several chromosomes, we investigated the possibility that they too evolved through segmental duplication events. We suggest that besides the transposition effect of the MER53 elements, the four copies of hsa-mir-1302-2 and the one copy of hsa-mir-1302-3 were produced through segmental duplication events. When Alu sequences are near or at the boundaries of the duplication units they are known to mediate the expansion of segmental duplications through recombination. Our results indicate that the hsa-miR-1302-2 and hsa-mir-1302-3 genes may have evolved because of Alu-mediated recombination events. However, this mechanism apparently does not apply to other members of the human miR-1302 family or to potential paralogs in the human genome. They may have evolved by the transposition of MER53 elements alone.

Small RNAs regulate gene expression in many ways: they mediate antiviral responses; they play a role in the organization of chromosomal domains; and they restrain the spread of selfish genetic elements. Small RNAs guide transcriptional and post-transcriptional silencing machinery to specific target sequences that include genes and transposable elements [51]. The target genes of miR-1302 are over-represented in functions that require the binding of metal ions and binding to DNA. They are mainly involved in metabolism, regulation of cellular physiological processes, signal transduction and transport. The MER53-derived miRNAs may, therefore, play important roles in cell proliferation and cell division, metabolism, development, pancreas physiology and pathology, nervous system physiology, diseases and in the immune response. Because the functions of miR-1302 have been predicted and predictions notoriously produce a large number of false-positives, a better method to assign a function to miR-1302 would be to combine the expression profiles of the miRNAs and the target genes. This is a study that we would like to do in the future.

## Conclusions

In this study, we report the origin and evolution of the miR-1302 family in the human genome. Overall, we have identified 36 novel potential paralogs of miR-1302 genes in the human genome and 58 orthologs of the human miR-1302 genes in 21 placental species. Our data show that all members of miR-1302 family are derived from MER53 elements and we have proposed that they emerged at the early stage of the recent 180 million years since eutherian mammals diverged from marsupials. Segmental duplication events have facilitated the expansion of the miR-1302 family while the expansion of these segmental duplications may also have been facilitated by Alu-Alu-mediated recombination events. Because, in placental species many miR-1302 genes have been gained and lost, we have proposed that their development proceeded according to the birth-and-death model of evolution. Furthermore, we have found that the predicted target genes of miR-1302 are over-represented in transportation, localization, and in system development processes as well as in the positive regulation of cellular processes. Many of the potential target genes are predicted to function in binding and transcription regulation.

## Methods

### Finding MER53-derived miRNAs Within Known Human miRNAs

The genomic locations and sequences of repetitive elements (including MER53) in the human genome were taken from the University of California Santa Cruz (UCSC) Genome Browser [52] using the Table Browser [53] and analyzed using RepeatMasker 3.27 [28]. The sequences and coordinates of the human pre-miRNAs and mature miRNAs were downloaded from miRBase v13.0 [27] and mapped to the human genome (hg18). The nomenclature for some members of the hsa-mir-1302 family differs between miRBase v13.0 and that in later releases. However, the sequences are the same and the new nomenclature has been explained in the Results section. The genomic locations of the miRNAs and the repeats in the whole genome sequence were compared using the UCSC Table Browser [53] and Galaxy [54]. If the overlap of coordinates (equivalent in this case to percentage identity) between a repetitive element and a pre-miRNA sequence was at least 50% for the pre-miRNA sequence or 100% for the mature miRNA sequence, then the miRNA was considered to be a repeat-derived miRNA [21]. To retrieve information and for further analysis, the data and results were processed using Linux shell and Perl scripts. Unless otherwise specified, all the data in the present work were analyzed using these tools.

### Genome-wide Identification of Potential Paralogs of MER53-derived miR-1302 Genes

To identify potential paralogs of MER53-derived miR-1302 genes, we developed a three-step operational scheme. We first searched the human genome for candidate MER53 elements using the BLAST program. Only hits with exact matches in the "seed region" (nucleotides 2-8) of miR-1302 were selected and two potential miR-1302 precursor sequences of length 110 nt harboring the "seed region" were excised from the hit sequences using a method similar to that described earlier [55]. An excised sequence refers to a mature miRNA that is processed from the left or right arm of a potential precursor sequence. Using MiPred [30], excised sequences that were predicted to be miRNA precursors were selected for further analysis. Finally, if the candidate sequence could be transcribed and if it was not the exon of a protein-coding gene, then it was assumed to be a miRNA precursor. To determine if the sequence was transcribed and was not an exon of a protein-coding gene, we compared the coordinates of the potential precursor sequences with the coordinates of ESTs and the exons of protein-coding genes in the hg18 genome assembly.

### Phylogenetic Distribution of the Orthologs of MER53-derived miR-1302 Genes

The orthologs of the eight members of the hsa-mir-1302 family in different organism were retrieved from the Multiz alignments of 44 vertebrate species [56]. When there were gaps or short inserts (1-10 bp) in the selected alignment region then the corresponding sequences were checked using the Ensembl Genome Browser. cja-mir-1302-5, ggo-mir-1302-6 and ggo-mir-1302-7 were detected by the BLAT program with best reciprocal hits. Because a number of workers have used the liftOver program provided by the UCSC Genome Bioinformatics Group to determine orthologs [57-61], we checked our previously determined ortholog sequences by applying liftOver to over.chain files (downloaded from ftp://hgdownload.cse.ucsc.edu/) to further authenticate them (see details in Additional File 3). The miPred [30] classifier was used to validate these sequences as potential pre-miRNAs.

### Evolution Analysis

The UCSC's phastCons program [62] was used to calculated the conservation score of each nucleotide of every member of the hsa-mir-1302 family of the 17-species Multiz alignment [56] from the UCSC Genome Browser [52]. The 17-species are human, chimp, rhesus, mouse, rat, rabbit, dog, cow, armadillo, elephant, tenrec, opossum, chicken, frog, zebrafish, tetraodon, pufferfish (fugu). The Multiz data contains a measure of evolutionary conservation for each nucleotide in the human genome against the other 16 genomes. To estimate the probability that each nucleotide belongs to a conserved element in the multiply aligned sequences, phastCons computes conservation scores based on a phylogenetic hidden Markov model [62]. The phastCons scores range from 0 to 1 and are a measure of the probability of purifying selection. The score estimates the probability of a nucleotide or a region being under selective pressure. The average score of each pre-miRNA was calculated from the per-site conservation score.

Sequences were aligned using the R-Coffee program [63]. Molecular evolutionary analyses were performed using MEGA 4 [64]. The extent of nucleotide sequence divergence was estimated using the uncorrected p-distances and evolutionary distances were calculated with the pairwise deletion option. Phylogenetic trees were reconstructed using the neighbor-joining method [65] and the statistical reliabilities of the internal branches were assessed using 1,000 bootstrap replicates.

Two independent approaches have been developed to detect segmental duplications (SDs) [66,67]. In one of the approaches, SDs (≥1 kp and ≥90% identity) were discovered by identifying high-copy repeats, removing the repeats from the whole genomic sequence and then searching for similar sequences in the genome. The repeat sequences were reinserted into the pairwise alignments, the ends of alignments were trimmed, and global alignments were generated (see reference [66] for details). To test our hypothesis that, in addition to the effect of MER53 transposition, SD events may contribute to the expansion of the hsa-mir-1302 family, we analyzed the segmental duplication data that were pre-computed by Bailey and his colleagues [66] and that we downloaded from the UCSC Genome Browser [52]. By comparing the coordinates, we determined whether human miR-1302 genes were located in regions of segmental duplications. We used Circos v0.52 [68] to show the relationships between segmental duplications harboring miR-1302 genes.

To find out if the segmental duplications that harbor miR-1302 genes are produced by Alu-mediated recombination events, we examined sequence features at the junctions of duplications. We defined junction sequences as the sequences at the terminal ends of segmental duplications spanning a ±5 bp interval. We compared the divergence of Alu elements located at the junctions with the divergence of the corresponding internal Alus in the pairing duplications [40]. Sequence divergence was estimated using Kimura's two-parameter model for genetic distance in MEGA 4 [64]. The data were analyzed using version 2.9.0 of the R programming language and environment for statistical computing and graphics [69].

### Target Prediction and Regulatory Function Analysis

We collected the 3'UTR-sequences of human coding refGenes from the UCSC Genome Browser [52]. Two programs, PITA [41] and TargetScan [42,43], were used to identify potential target sites of hsa-miR-1302. The intersection of the two independently computed sets of target genes was used to list the valid potential targets. The number of possible target sites for human miR-1302 in all human coding genes was calculated after removing the coordinates of overlapping genes. To determine the functional categories to which the target genes belong, we used the WebGestalt program [44] to display the GO categories of the target genes. We also mapped the genes to KEGG pathways [45].

## Abbreviations

MicroRNA: miRNA; GO: Gene Ontology; KEGG: Kyoto Encyclopedia of Genes and Genomes

## Authors' contributions

ZY and XS both conceived of the study and participated in its design. ZY performed the computational experiments and drafted the manuscript. XS refined the manuscript. DJ and YD helped to analyze data. ZL, LG, HL and JX contributed to discussions on the evolutionary analyses and helped to draft the manuscript. All authors read and approved the final manuscript.

## Supplementary Material

Additional file 1 **Supplementary figures**. Figures of the distribution of members of hsa-mir-1302 family on the human chromosomes; Alignments of two MER53 elements and corresponding derived miRNA genes; Distribution relationship between segmental duplications (SD) and Alus and miR-1302 genes in the human genome. Click here for file

Additional File 2 **Genome-wide Identification by MiPred of Potential Paralogs of MER53-derived miR-1302 Genes**. The rows with grey backgrounds are already known precursors of hsa-miR-1302 that were found to be derived from MER53 elements. Click here for file

Additional File 3 **The sequences of orthologs of the human miR-1302 family in 21 placental mammals**. The list includes 58 orthologs of the human miR-1302 family in 21 placental species predicted as pre-miRNAs by computational method. Click here for file

Additional file 4 **Estimates of Evolutionary Divergence between Sequences**. Pairwise p- distances for the 58 orthologs of the human miR-1302 family in 21 placental species. All positions containing alignment gaps and missing data only were eliminated in pairwise sequence comparisons (Pairwise deletion option) leaving a total of 221 positions in the final dataset. Click here for file

Additional file 5 **miRNA and its neighboring genes in the duplication pairs**. Segmental duplication pairs, duplication coordinates and the annotated genes and miRNAs in the SD are listed. Click here for file

Additional file 6 **Targets and target sites of miR-1302 predicted by PITA in the human genome**. 17,844 targets and 39,154 target sites predicted by PITA in human genome. The predicted scores are also provided. Click here for file

Additional file 7 **Targets, conserved sites and poorly conserved sites of miR-1302 predicted by TargetScan**. 2,055 targets, with a total of 94 conserved sites and 2,334 poorly conserved sites predicted by TargetScan are listed. Click here for file

Additional file 8 **Common target lists of PITA and TargetScan**. Input_id is the common target gene symbol for genes in the target lists of PITA and TargetScan, Locus link id, symbol, gene_name, gene_stable_id are the attributes of the known genes of the input_ids in WebGestalt. Click here for file

Additional file 9 **Conserved targets in the common target lists predicted by TargetScan and PITA**. The data sets are the intersection sets of the target lists predicted by TargetScan and PITA. Click here for file

Additional file 10 **The KEGG Table organizes genes based on the KEGG biochemical pathways**. The KEGG Table shows KEGG pathways associated with the gene set (column 1), the number of genes in each pathway (column 2) and the Entrez Gene IDs for the genes (column 3). The 4th column gives the parameters for the enrichment of the KEGG pathway. Click here for file
