# Supplementary material for: Origin and evolution of a placental-specific microRNA family in the human genome
Source: BMC Evol Biol. 2010 Nov 10;10:346. doi: 10.1186/1471-2148-10-346 (PMC2996404; doi:10.1186/1471-2148-10-346)

**FIGURE LEGENDS**

**Figure S1** The distribution of members of the hsa-mir-1302 family on the human chromosomes.

(A) hsa-mir-1302-1 (B) hsa-mir-1302-2 (C) hsa-mir-1302-3 (D) hsa-mir-1302-4 (E) hsa-mir-1302-5 (F) hsa-mir-1302-6 (G) hsa-mir-1302-7 (H) hsa-mir-1302-8

**Figure S2** Phylogenetic tree of 58 orthologous sequences of the human miR-1302 family in 21 placental species. The numbers at the node of each branch is the percent bootstrap value (1000 resamplings). Only values >50% are shown. We have added the genome assemble version (such as hg18) to each miRNA gene designation.

**Figure S3** (A) The pairwise alignment of hsa-mir-1302-2 and hsa-mir-1302-3. (B) The pairwise alignment of MER53 elements from which hsa-mir-1302-2 and hsa-mir-1302-3 are derived.

**Figure S4** Distribution relationship between segmental duplications (SD), Alus and miR-1302 genes in the human genome.

1. From top to bottom:

The coordinates of SDs on chromosome 1

chr1 466–30596(-)

chr1 487–30596(+)

chr1 487–76975(-)

chr1 8257–76975(+)

Alu repeats

hsa-mir-1302-2

Genes

1. From top to bottom:

The coordinates of SDs on chromosome 2

chr2 113887481–114076781(-)

chr2 114046769–114069083(-)

chr2 114046769–114076456(-)

chr2 114046769–114076720(+)

Alu repeats

hsa-mir-1302-3

Genes

1. From top to bottom:

The coordinates of SDs on chromosome 9

chr9 413– 193762(-)

chr9 437– 30515(-)

chr9 845– 30515(+)

chr9 8507–30515(+)

Alu repeats

hsa-mir-1302-2

Genes

1. From top to bottom:

The coordinates of SDs on chromosome 15

chr15 100218756–100330295(-)

chr15 100263880–100338121(-)

chr15 100307957–100338402(+)

chr15 100307957–100338529(-)

Alu repeats

hsa-mir-1302-2

Genes

1. From top to bottom:

The coordinates of SDs on chromosome chr19

chr19 11002–123445(-)

chr19 11002–33344(-)

chr19 11002–33344(+)

chr19 11002–79672(+)

Alu repeats

hsa-mir-1302-2

Genes

**Figure S5** The Gene Ontology (GO) results for the predicted target genes of hsa-mir-1302. Chromosome distribution, tissue expression pattern, cellular component, molecular function and biological progress were analyzed using the WebGestalt program. (A) Chromosome Distribution: Each chromosome is represented by a bar. Each gene is represented by a red cross indicating its location on the chromosome. (B) Tissue Expression Pattern: Each tissue is represented by a bar. The height of the bar represents the number of genes that are expressed in the tissue. (C–E) Three separate DAGs for the different GO ontologies: Cellular Component (C), Biological Process (D) and Molecular Function (E). Each GO term is a node in the DAG. The enriched GO categories are indicated in red (the top 10 have a adjusted p values of < 0.05) and their non-enriched parents are in black. For each enriched category, the name of the GO term, the number of genes in the category and the adjusted p value indicating the significance of enrichment, are given.

**Figure S1**

A) hsa-mir-1302-1 on chr12


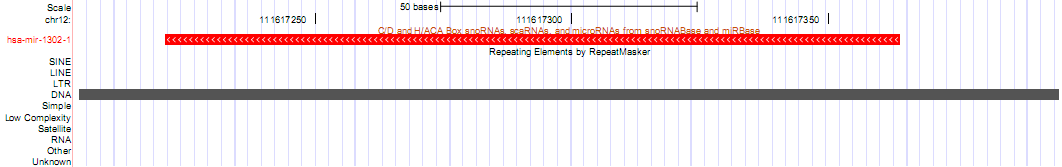


B) hsa-mir-1302-2 on chr1


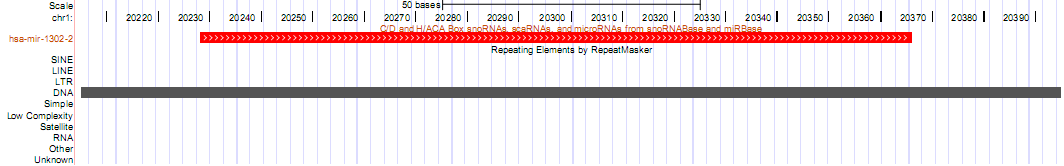


hsa-mir-1302-2 on chr9


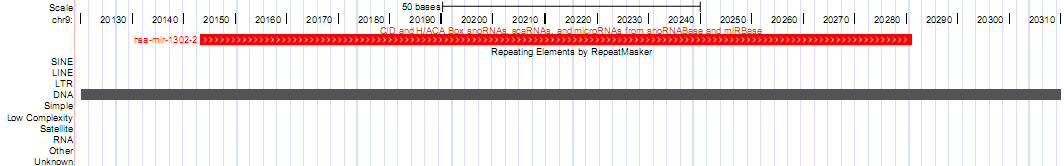


hsa-mir-1302-2 on chr15


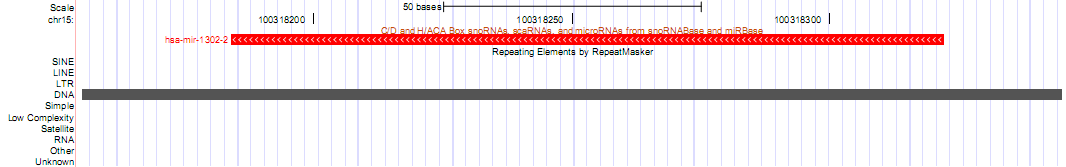


hsa-mir-1302-2 on chr19


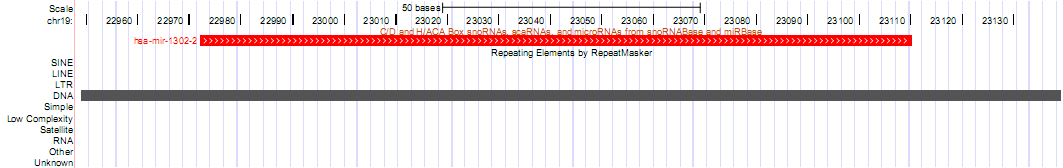


C) hsa-mir-1302-3 on chr2


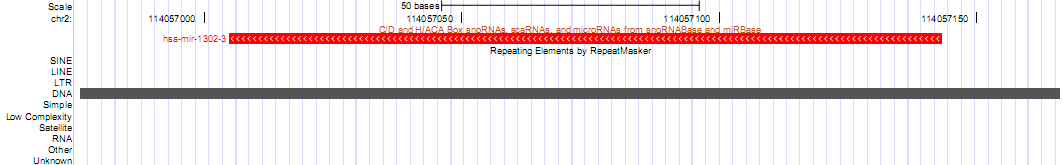


D) hsa-mir-1302-4 on chr2


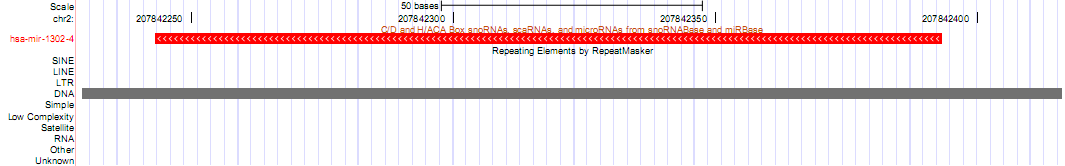


E) hsa-mir-1302-5 on chr20


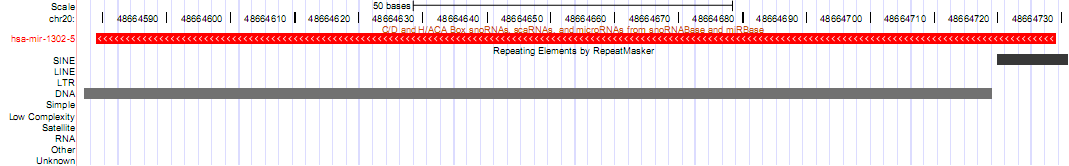


F) hsa-mir-1302-6 on chr7


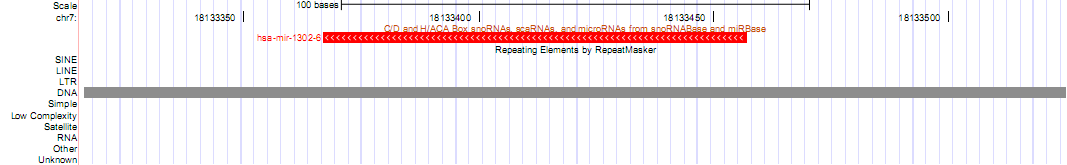


G) hsa-mir-1302-7 on chr8


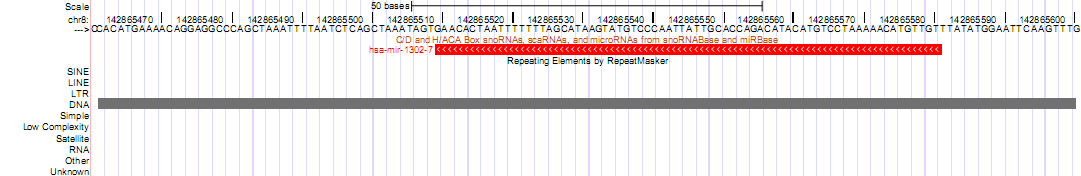


H) hsa-mir-1302-8 on chr9


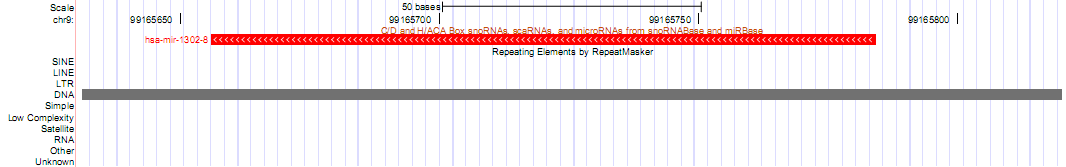


**Figure S2**

**Figure S3**

A)


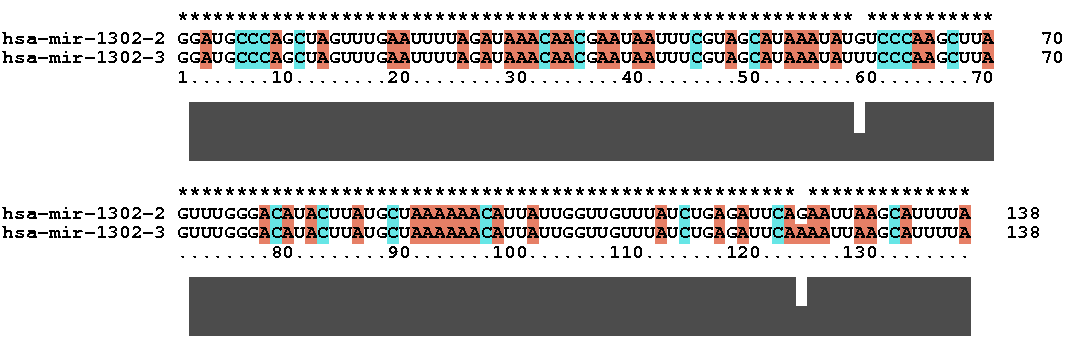


B)


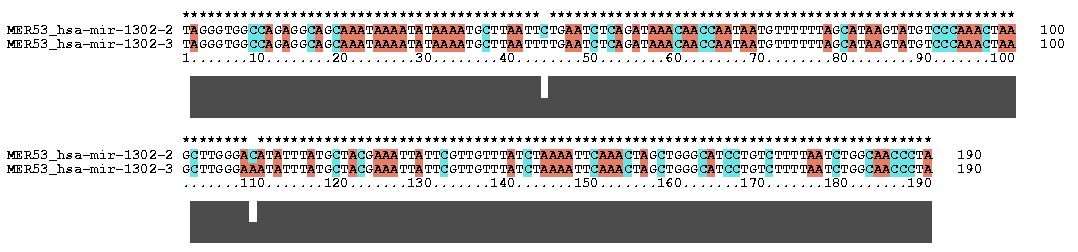


**Figure S4**

**A) chr1**

**
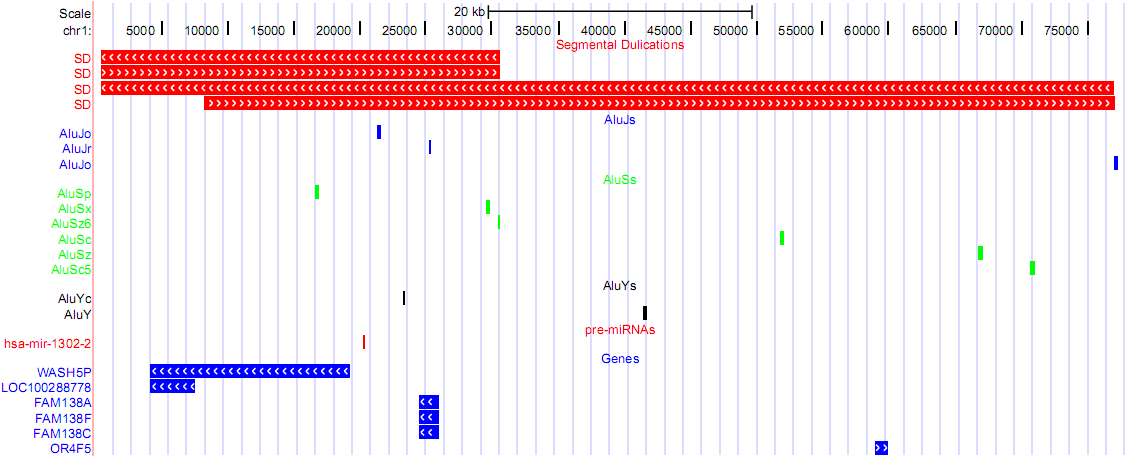
**

**B) chr2**

**
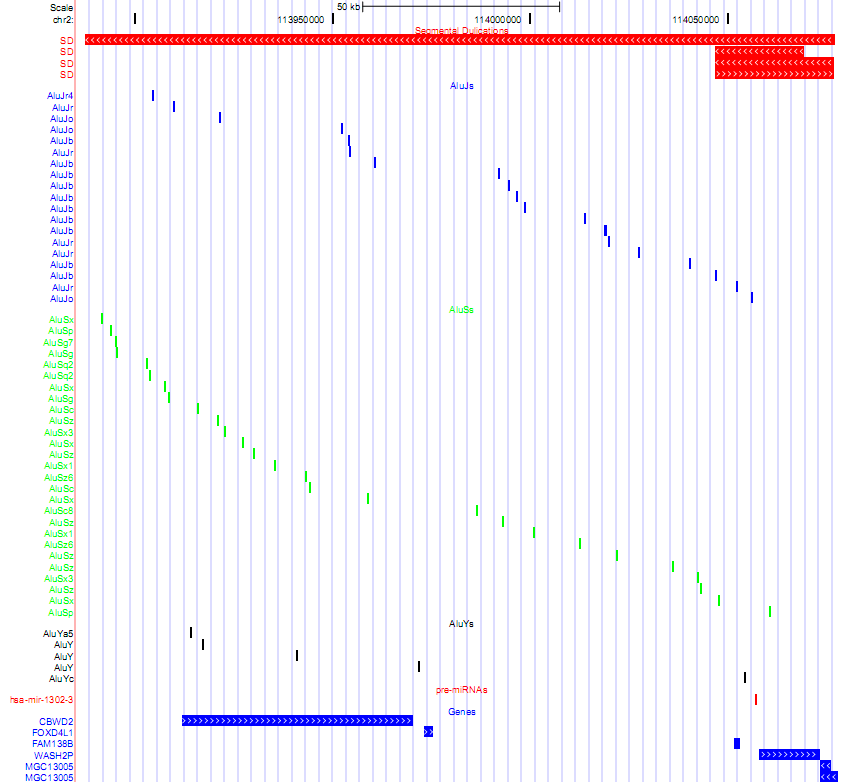
**

**C) chr9**


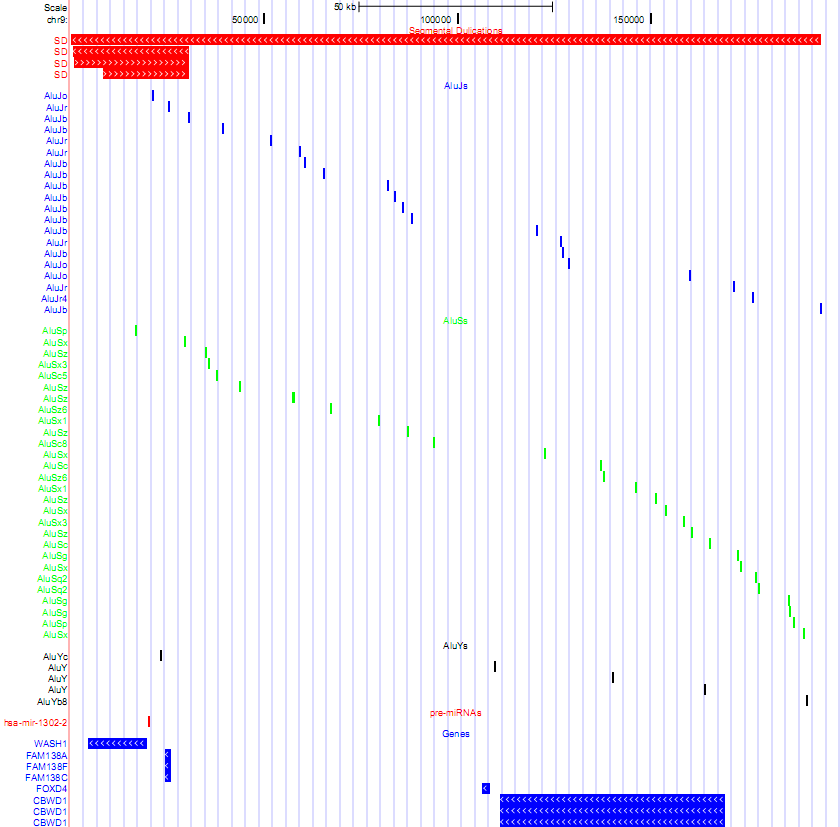


**D) chr15**


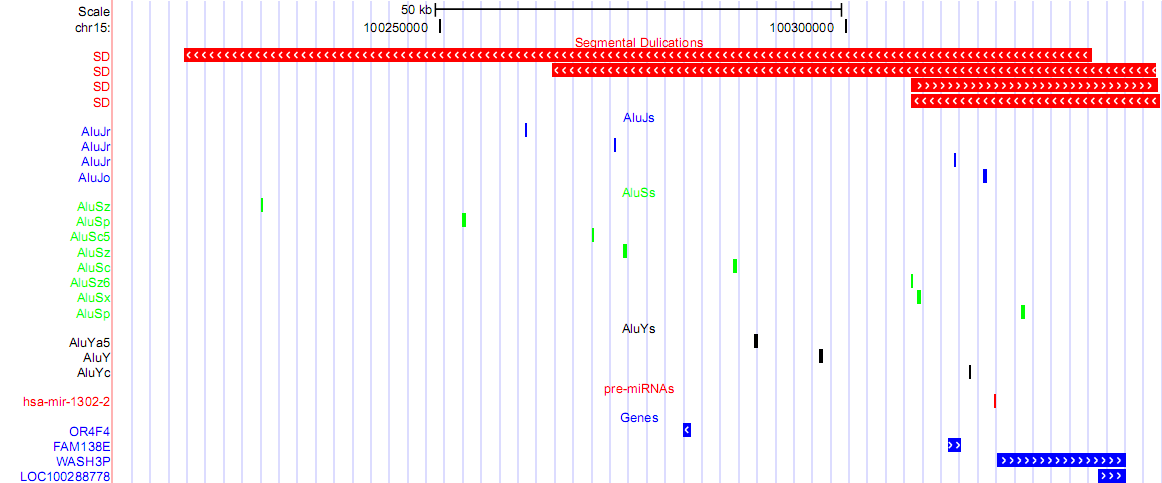


**E) chr19**

**
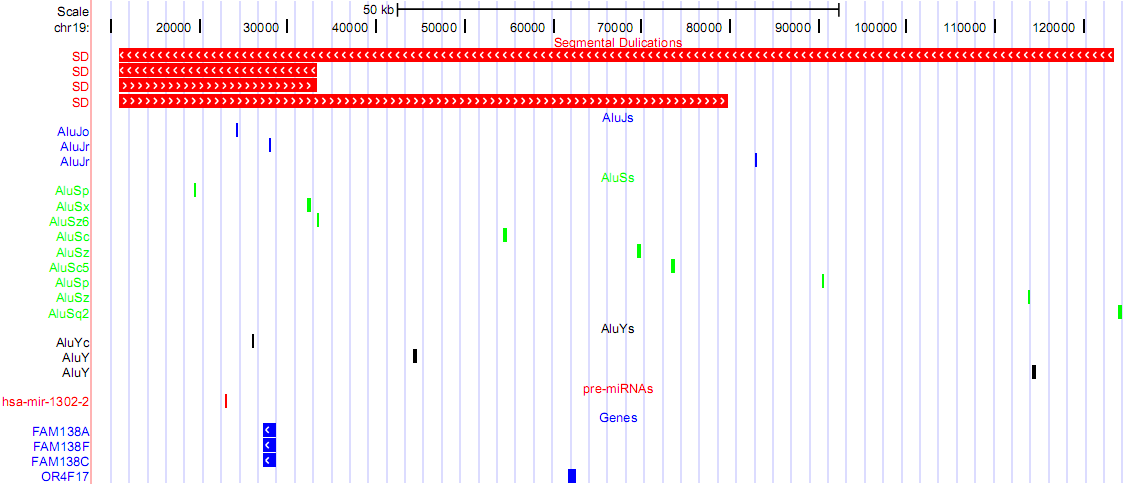
**

**Figure S5**

A) Chromosome distribution


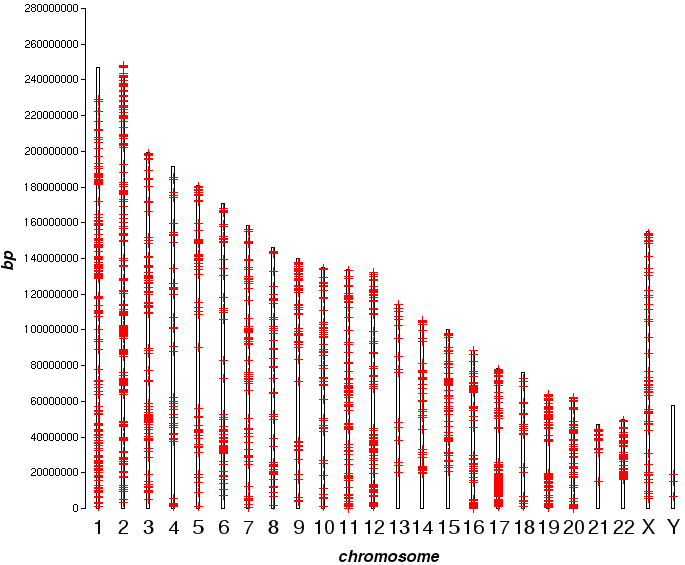


B) Tissue expression pattern


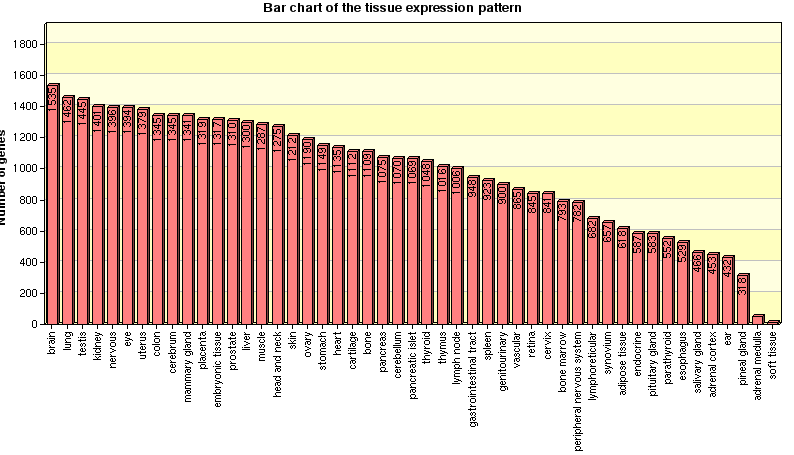


C) Cellular Component


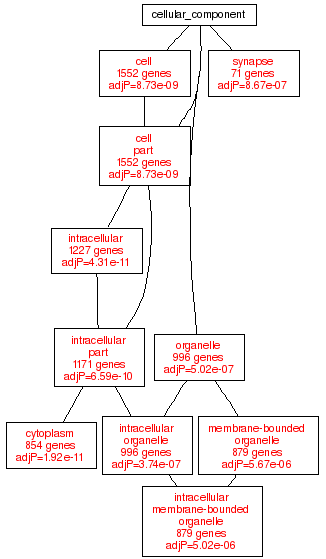


D) Biological Process


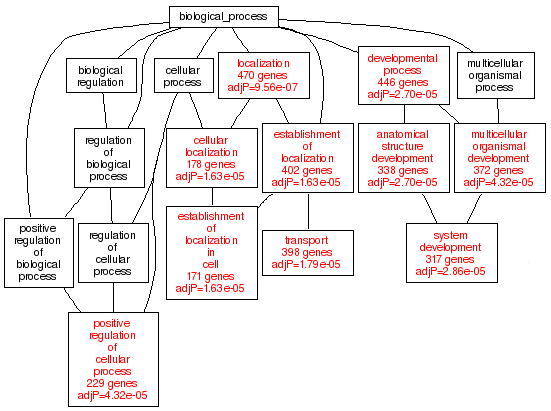


E) Molecular Function


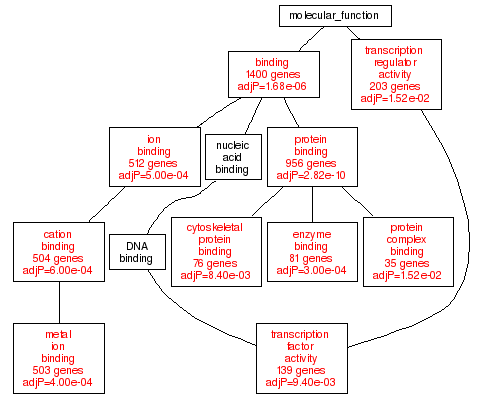

Supplement: Additional file 1 — Supplementary figures. Figures of the distribution of members of hsa-mir-1302 family on the human chromosomes; Alignments of two MER53 elements and corresponding derived miRNA genes; Distribution relationship between segmental duplications (SD) and Alus and miR-1302 genes in the human genome. [file 1471-2148-10-346-S1.DOC]
