# Supplementary material for: Origin and evolution of a placental-specific microRNA family in the human genome
Source: BMC Evol Biol. 2010 Nov 10;10:346. doi: 10.1186/1471-2148-10-346 (PMC2996404; doi:10.1186/1471-2148-10-346)
Supplement: Additional File 3 — The sequences of orthologs of the human miR-1302 family in 21 placental mammals. The list includes 58 orthologs of the human miR-1302 family in 21 placental species predicted as pre-miRNAs by computational method. [file 1471-2148-10-346-S3.DOC]

Additional File 3

We obtained all the precursor sequences of the human miR-1302 family from miRBase v13.0 [1]. These sequences were then submitted to the UCSC human genome (hg18) browser [2] using the sequence alignment tool BLAT[3]. Using each of the human miR-1302 sequences as the reference sequence, we retrieved their homologs and genome coordinates from the MULTIZ multiple alignment files. If there were gaps or short inserts (1–10 bp) in the selected alignment region then the corresponding sequences were checked using the Ensembl Genome Browser. The miPred [4] classifier was used to validate these sequences as potential pre-miRNAs.

Because a number of workers have used the liftOver program provided by the UCSC Genome Bioinformatics Group to determine orthologs [5–9], we checked the previously determined ortholog sequences (please see the sequences in the following part) by applying liftOver to over.chain files (downloaded from <ftp://hgdownload.cse.ucsc.edu/>). We found that only the calJac1-mir-1302-5, gorGor3-mir-1302-6 and gorGor3-mir-1302-7 orthologs were not in the liftOver results. We can show, however, that these othorlogs were detected by the BLAT program with best reciprocal hits.

(1) calJac1-mir-1302-5

>calJac1-mir-1302-5_contig6037:61050–61188(-)

UGCCUGGCCUCUCAUUAAAUUUGAUUUUCAGACACAUUACAAAUUUGUUUAGUAUUAGUGUAUCCCAUGUAAUCUUUGGGACAUACGCUAAAAUAAUUGUUCCUUGUUUACUGAAAAUUUUAAUUAGGUGUCCUGUAUU

The best hit of the sequence of calJac1-mir-1302-5 blats Human Mar. 2006 (NCBI36/hg18) Assembly in the UCSC Genome Browser:

<http://genome.ucsc.edu/cgi-bin/hgTracks?position=chr20:48664580-48664729&db=hg18&ss=../trash/hgSs/hgSs_genome_5fc6_ab2b70.pslx+../trash/hgSs/hgSs_genome_5fc6_ab2b70.fa&hgsid=170006797>

The best hit of the sequence of hsa-mir-1302-5 blats Marmoset June 2007 (WUGSC 2.0.2/calJac1) Assembly in the UCSC Genome Browser:

<http://genome.ucsc.edu/cgi-bin/hgTracks?position=Contig6037:61050-61188&db=calJac1&ss=../trash/hgSs/hgSs_genome_5fce_ab2d60.pslx+../trash/hgSs/hgSs_genome_5fce_ab2d60.fa&hgsid=170025794>

These results indicate that the mir-1302-5 gene is in an intron of the orthologous regions of FAM65C of both human and marmoset.

(2) gorGor3-mir-1302-6 and gorGor3-mir-1302-7

There are gaps in the regions of the corresponding ortholog blocks of hsa-mir-1302-6 and hsa-mir-1302-7 in the first daft assembly of the gorilla genome, the only one provided by UCSC Genome Browser. However, the third draft assembly (gorGor3) is available on the Ensembl Browser (<http://www.ensembl.org/Gorilla_gorilla/Info/Index/>). We, therefore, determined the orthologs of these two miRNAs using the BLAT program with best reciprocal hits in the UCSC Genome Browser (human) and in the Ensembl Browser (gorGor3).

>gorGor3-mir-1302-6_chr7:18319759–18319848(+)

AACAAAUAAUUUGGUAAUAUAUGUAUGGCCCACACAAUAUUUAGGACAACAAUAUUUGGGACAUACUUAUGCUAAAAAAGUAUUUGUUGA

The best hit of the sequence of gorGor3-mir-1302-6 blats Human Mar. 2006 (NCBI36/hg18) Assembly in the UCSC Genome Browser:

<http://genome.ucsc.edu/cgi-bin/hgTracks?position=chr7:18133368-18133457&db=hg18&ss=../trash/hgSs/hgSs_genome_5fef_9c9c30.pslx+../trash/hgSs/hgSs_genome_5fef_9c9c30.fa&hgsid=170006797>

or, when zoomed out

<http://genome.ucsc.edu/cgi-bin/hgTracks?insideX=118&revCmplDisp=0&hgsid=170006797&hgt_doJsCommand=&hgt.out2=+3x+&position=chr7%3A17983413-18283412&hgtgroup_map_close=0&hgtgroup_phenDis_close=1&hgtgroup_genes_close=0&hgtgroup_rna_close=0&hgtgroup_expression_close=0&hgtgroup_regulation_close=0&hgtgroup_compGeno_close=0&hgtgroup_neandertal_close=0&hgtgroup_varRep_close=0&hgtgroup_encodeGenes_close=1&hgtgroup_encodeTxLevels_close=1&hgtgroup_encodeChip_close=1&hgtgroup_encodeChrom_close=1&hgtgroup_encodeCompAndVar_close=1>

The best hit of the sequence of hsa-mir-1302-6 blats gorGor3 in Ensembl Browser. We found the syntenic genes, SNX13 and PRPS1L1, in both the human and gorilla genome:

<http://www.ensembl.org/Gorilla_gorilla/blastview/BLA_cSPEnICHT>

or

<http://www.ensembl.org/Gorilla_gorilla/Location/View?r=7:18317759-18321848;h=BLAST_NEW:BLA_cSPEnICHT!!20100923>

>gorGor3-mir-1302-7_chr8:142260329-142260400(-)

ACAACAUGUUUUUAGGACAUGUAUGUCUGGUGCAAUAAUUGGGACAUACUUAUGCUAAAAAAAUUAGUGUUC

The best hit of the sequence of gorGor3-mir-1302-7 blats Human Mar. 2006 (NCBI36/hg18) Assembly in the UCSC Genome Browser. We could not identify syntenic genes in the 500 kb centered on the mir-1302-7 gene.

<http://genome.ucsc.edu/cgi-bin/hgTracks?position=chr8:142865510-142865581&db=hg18&ss=../trash/hgSs/hgSs_genome_603e_9ca5a0.pslx+../trash/hgSs/hgSs_genome_603e_9ca5a0.fa&hgsid=170006797>

The best hit of the sequence of hsa-mir-1302-7 blats gorGor3 in Ensembl Browser:

<http://www.ensembl.org/Gorilla_gorilla/blastview/BLA_Or542bVIi>

or

<http://www.ensembl.org/Gorilla_gorilla/Location/View?r=8:142258329-142262400;h=BLAST_NEW:BLA_Or542bVIi!!20100923>

**In summary, we successfully validated the 58 sequences as orthologs of the human miR-1302 family.** Although some sequences have diverged substantially from the corresponding orthologs of human (e.g. bosTau4-mir-1302-1), we have tentatively named them after their human orthologs. We have built FASTA files for each of the 58 orthologous sequences of the human miR-1302 family in 21 placental species The initial line describes the sequence and provides information as follows (the first FASTA file as the example):

**bosTau4-mir-1302-1_chr17:64804303-64804446(+)**: the bovine ortholog of hsa-mir-1302-1

**bosTau4**: the UCSC version of the cow genome assemble

**chr17:64804303-64804446(+)**: the coordinates of the sequence in the cow genome.

>bosTau4-mir-1302-1_chr17:64804303-64804446(+)

CAAGAUGCCUAGUUAAAAGUAGACUUCAGAUAAACAGUGAAUAGGGUGGGAUUCUUUUUUUUUAUAAGGAUAACCCAUAUAAAAUUUACACUAAAAAAUUAUUCAUUGUUUAUCUGAAAUUCAAAUGUAACAAGGAGUCCUGUG

>bosTau4-mir-1302-2_chr5:113887806-113887956(+)

AGAUGCCAAGGUCAUUUGAAUUUCAGAUGAAAAAAAAAGUUUUUUAUUUUUAUUUUUAAUAUAAGUAUGUCCCAAACCGAGCAUGGGGCACACUUACAUUAAAAAAAAAAAUCAUUUGUUGUUUAUUUGAAAUUCAGGAUUAAGUGUUUUG

>bosTau4-mir-1302-6_chr4:27889431-27889502(-)

CAAAUAUUUUCAAUAUAAAUAUGUCCCUGAAAUAUUUGGGACAUACUUGUAAUGAAAAAACUUAUUUGUUGA

>calJac1-mir-1302-1_Contig471:681564-681705(+)

CAGAAAGCCCAGAUAAAUUUGGAUUUCAAGUAAACAAUCAAUAAUUGUGUAUGUAAGUAUAUCCCAUACAAUAUUUGGGACAUACUUGUGCUAAAAAUUAUUCUUUGCUUAUCUGAAAUUCAAAUGUAACUAGAUUCCUGUA

>calJac1-mir-1302-2_Contig11097:45387-45524(+)

GGAUGCCCGGUGACUUUGAAUUUCAGAUAAAGAGCGAACAAUUUCUUAGCACAGAUAUGACCCAAGCUUAGUUUGGGACAGACUUAUGCUAAAAAAUAUUAUUGGUUGUUUAUCUGAAAUUCAAAAUUAACCAUUUUA

>calJac1-mir-1302-4_contig194:788058-788207(-)

AAUGCAGAAGCACAGCUAAAAUUUGAGUUUCAGAUAAACAAAUUUUUCUUAGAAUAAGUAUAUCUCCAUGCAACAUUUGGGACAUACUUAUGCUAAAACAUUAUUUGUGUUUCAUCUGAAAUUCAAAUUCAACUGGAUAUCCUGUAUUUU

>calJac1-mir-1302-5_contig6037:61050-61188(-)

UGCCUGGCCUCUCAUUAAAUUUGAUUUUCAGACACAUUACAAAUUUGUUUAGUAUUAGUGUAUCCCAUGUAAUCUUUGGGACAUACGCUAAAAUAAUUGUUCCUUGUUUACUGAAAAUUUUAAUUAGGUGUCCUGUAUU

>calJac1-mir-1302-6_contig379:58810-58899(-)

AACAAAUAAUUUGGUAAUAUAUGUAUGCCCCACAAAAUAUUUAAGACAGCAAUAUUUGGGACAUACUUAUGCUAAAAAAGUAUUUGUUGA

>calJac1-mir-1302-7_contig7680:7431-7500(-)

GCAACAUUUUUUAGUACAUGUAUGUCUGGUACAGUAAUUGGGACAUACUUAUGCUAAAAAAUUAGUAUUC

>calJac1-mir-1302-8_contig67:732720-732850(-)

CCCAUUUAAACCUGCAUUUCAUAUUAACAGAGUGAUUUUCAGCAUUAGUAUAUCAUGUGCAGUAUUUAGGAUAUAUUUAUGCUAAAAAAAAAUUAGGUGGUGUUGAUCUGAAAUUCCAGUGUAGAUGGGUG

>cavPor3-mir-1302-1_scaffold_115:3780013-3780155(-)

CAGAUCCUCGGUUACCUCUGCGUUUCAGCUAAACAAUGAAUGAUUUUGUGUAGAAAGCCUACGCCACACACUAUUGGGGACAUGCUUGUAAACAAAAUCCUUUGAUUGUUUAGCUGAAAUUCAAAUGUAACUAGAAGUCUGUA

>choHof1-mir-1302-1_scaffold_19898:11584-11706(-)

CAAAACUUUCAGAUAAACAAUGAAAAAAUGUUGUAUAAGUAUGUCCCACGCAGUAUUUGGGGUAUGCCUAUACUAAAAAAAUUAUUGUUUAUCUGAAAUUCAAAUGUAACUUAGAGACAAAUA

>choHof1-mir-1302-4_scaffold_74222:4954-5100(-)

AAUACAGGAGCCCAACUGAAUUUGAAUUCAGAUAAACAAUUUUUUCUUAGGAUAAGUAUGUCCUAUGUAAUAUUUGAGACCUACUUAUACUAGAAAUUAUUUGUGCUUUAGCUGAAAUUCAGAUUUAACUGGACAUCCUGUAUUCU

>equCab2-mir-1302-1_chr8:19720304-19720445(+)

GGAUGCCCAGUUAUAUUUGGAUUUCAGAUAAGCAAUGAAUAGGUUUUUGUAUAAGUAUAGCCCAUGCAAUAUUUGGGACAUACUUAUACUAAAAAGUUAUUCAUCGUUUAUCUGAAAUUCAAAUGUAACUGGGAGUCUUGCA

>equCab2-mir-1302-2_chr6:30590497-30590646(-)

GGAUGCUAAGUUAAUGUGAAUUUCAGAUAAAAAGCAAAGAUAGAUAGAUAGAUAUAGUAUAAGUAUGUCCCAAACUAAGCGUGGGGCAUAUUUAUACUAAAAAAUUAUUUAUUGUUCAUUUUUUAAAUUCAAAAUUAACUAGUUGUUUGG

>equCab2-mir-1302-4_chr18:81367490-81367627(-)

AAUGCAGAAACACAGCUAACUCAGAAUUUUAGACAAAUAUUUUUUAAGAAUAAGUAUGUCCCGUGCAAUAUUGGGGACAUACUUAUGCUGACACUUAAUUUGCGUUUCCUCUGAAAUUCAACUUUAGUCCUGUAUCUU

>equCab2-mir-1302-8_chr25:3535134-3535263(-)

CCCAGUUAAAUUUGAAUUUCACAAAGGCAACGAGCAAUUUUUGGUCUUAAGUAUGUCCCAUGCAAUAUUUGGGACACAUUUGUACUAAAAAUAUUAUGUGUUGUUUAUCUGAAAUUCACAUUUAACUGGG

>felCat3-mir-1302-1_scaffold_140836:36457-36608(+)

AAGGAUGCCCGGCUACAUUUGAAUUUCAGACAAACAAUGAAUAGAUUUUCUUGUCUAACCAUAACCCAUGCGAUAUUCGGGACAUACUUACAUUGGACUAAAAAAUUACUCACUGGGUGUCUGAAAUUCAAAUGCAACAGGGGAAUUCUGUA

>gorGor1-mir-1302-1_Supercontig_0140050:628-770(+)

CAGAAAGCCCAGUUAAAUUUGAAUUUCAAGUAAACAAUGAAUAAUUGUGUAUGUAAGAAUAUCCCAUACAAUAUUUGGGACAUACUUAUGCUAAAAAUUAUUCCUUGCUUAUCUGAAAUUCAAAUGUAACUAGGAUUCCUGUA

>gorGor1-mir-1302-2_Supercontig_0000035:132014-132151(-)

GGAUGCCCAGUUAGUUUGAAUUUUAGAUAAACAACGAAUAAUUUCGUAGCAUAAAUAUGUCCCAAGCUUAGUUUGGGACAUACUUAUGCUAAAAAACAUUAUUGGUUGUUUAUCUGAGAUUCAAAAUUCAGCAUUUUA

>gorGor1-mir-1302-4_Supercontig_0002083:36376-36526(-)

AAUGCAGAAGCACAGCUAAAAUUUGAAUUUUAGAUAAACAAAUUUUUCUUAGAAUAAGUAUGUUCCCAUGCAACAUUUGGGACAUACUUAUGCUAAAAUAUUAUUUGUGUUUCAUCUGAAAUUCAAAUUCAACUGGACAUCCUGUAUUAU

>gorGor1-mir-1302-5_Supercontig_0174107:1341-1490(-)

UGCCCGGCCUCCCAUUAAAUUGGUUUUUCAGACAAAUCACAAAUUUGUUUAGGUAUAAGUAUAUCCCAUGUAAUCUUUGGGACAUACUUAUGCUAAAAUAAUUGUUCCUUGUUGAUUGGAAAUCUUAAUUUUAAUUAGGUGUCCUGUAUU

>gorGor1-mir-1302-8_Supercontig_0001028:128236-128363(-)

CCAAUUUAAACUUGAAUUUCAUAUAAACACCGUAAUUUUCAGCAUUAGUGUAUCACAUGCAGUAUUUGGGACAUACUUAUGCUAAAAAAUUAGGUGGUGUUGAUCUGAAAUUCCAGUGUAGAUGGGCA

>gorGor3-mir-1302-6_chr7:18319759-18319848(+)

AACAAAUAAUUUGGUAAUAUAUGUAUGGCCCACACAAUAUUUAGGACAACAAUAUUUGGGACAUACUUAUGCUAAAAAAGUAUUUGUUGA

>gorGor3-mir-1302-7_chr8:142260329-142260400(-)

ACAACAUGUUUUUAGGACAUGUAUGUCUGGUGCAAUAAUUGGGACAUACUUAUGCUAAAAAAAUUAGUGUUC

>loxAfr2-mir-1302-2_scaffold_35367:1182-1312(+)

GAUAGUUCCCAUUAAAUUUGAAUUUCUGGUAACUAACAAUUUUUUGUUUAAGUAUGUCCUAAAUCAUGCAUGGGUCAUACUUAUGCUAACAAAAUAGUCUUUAUCUGAAAUUCAAAUGUAACUAAGUGUAU

>micMur1-mir-1302-1_scaffold_6025:69793-69935(-)

CAUAAUGCCCAGUUAAAUUUGAAUUUCAGGCACACAAUGAAUAAUUGUGUGUAUAAGUAUAUCCCAUACAAUAGUUGGGAUAUACUUAUACUAAAAAUCAGUCAUUGAUUCUCUGAAAUUCAAAUGUAAGUGAGAUUCUUAUA

>micMur1-mir-1302-4_scaffold_244:429180-429329(-)

AAUGCAGAAGCACAGCUAAAUUUGAGUUUCAGAUGAACAAUCAAUUUUUUAAGAAUAAGUAUGUCCCAUGCAAUAUUUGGGACUUACUUAUGCUAGACCAUUAUUUGUAUUUCAUCUGAAAUUCAAAUUUAACUGGACAUCUGUAUUUU

>micMur1-mir-1302-6_scaffold_859:139758-139832

AACAAAUAAUUUUGGAGUAUGACCAUGUGUCAUGCAAUAUUUGGUACAUACUCAUUCUAAGCAAUUAUUUGUUGA

>myoLuc1-mir-1302-8_scaffold_145797:66528-66654(-)

CCCAUUUAAACUUGAAUUUCAUAUAAGCGACAAAUGAUUUUUUUAGUGUAUUUCUCAUGCUAUAUUUGGGACAUACUUACACUAAAAAUAUUACAUGUUUUUCUGAAACUCAAAUUUAACUCCCGCA

>oryCun1-mir-1302-8_scaffold_14457:2166-2280(+)

CCUAGUUAAACCCGAAUUUCAUACAAGCAACAAGUGAUUUUUAGUACAGGCAAUGUUCAGGCCAUAUGUGUACUAAAAAUAGGUGUUAUUGAUCUGAAAUUGCAGUUGAACUGGC

>otoGar1-mir-1302-1_scaffold_101867.1-188945:109890-110032(-)

UAUAAUGCCCAGUUAAAUUUGAAUUUCAGGUAAACAAUGAAUAAUUGUAUAUGUAAGUAUAUCCCAUACAAUAUUUGGGAUAUACUUAUAUUAAAAAUUGGUAAUUGCUUAUCUGAAAUUCAAAUGUAAGUGGGAUUCUUAUA

>panTro2-mir-1302-1_chr12:114060674-114060816(-)

CAGAAAGCCCAGUUAAAUUUGAAUUUCAAGUAAACAAUGAAUAAUUGUGUAUGUAAGAAUAUCCCAUACAAUAUUUGGGACAUACUUAUGCUAAAAAUUAUUCCUUGCUUAUCUGAAAUUCAAAUGUAACUAGGAUUCCUGUA

>panTro2-mir-1302-2_chrUn:1512127-1512264(-)

GGAUGCCCAGUUAGUUUGAAUUUUAGAUAAACAACGAAUAAUUUCGUAGCAUAAAUAUGUCCCAAGCUUAGUUUGGGACAUACUUAUGCUAAAAAACAUUAUUGGUUGUUUAUCUGAGAUUCAAAAUUAAGCAUUUUA

>panTro2-mir-1302-4_chr2b:212856670-212856818(-)

AAUGCAGAAGCACAGCUAAAAUUUGAAUUUCAGAUAAACAAAUUUUUCUUAGAGUAAGUAUGUCCCCAUGCAAUAUUUGGACAUACUUAUGCUAAAAUAUUAUUUGUGUUUCAUCUGAAAUUCAAAUUCAACUGGACAUCCUGUAUUUU

>panTro2-mir-1302-6_chr7:18299715-18299804(-)

AACAAAUAAUUUGGUAAUAUAUGUAUGGCCCACACAAUAUUUAGGACAACAAUAUUUGGGACAUACUUAUGCUAAAAAAGUAUUUGUUGA

>panTro2-mir-1302-7_chr8:141630266-141630337(-)

ACAACAUGUUUUUAGUACAUGUAUGUCUGGUGCAAUAAUUGGGACAUACUUAUGCUAAAAAAAUUAGUGUUC

>panTro2-mir-1302-8_chr9:96558860-96558987(-)

CCCAUUUAAACUUGAAUUUCAUAUAAACACCGUAAUUUUCAGCAUUAGUGUAUCACAUGCAGUAUUUGGGACAUACUUAUGCUAAAAAAUUAGGUGGUGUUGAUCUGAAAUUCCAGUGUAGAUGGGCA

>ponAbe2-mir-1302-2_chr2b:21154807-21154944(+)

GGAUGCCCAGUUAGUUUGAAUUUUAGAUAAACAACGAAUAAUUUCUUAGCCUAAAUAUGUCCCAAGCUUAGUUUGGGACAUACUUAUGCUAAAAAACAUUAUUGGUUGUUUAUCUGAGAUUCAAAAUUAAGCAUUUUG

>ponAbe2-mir-1302-4_chr2b:98768701-98768850(-)

AAUGCAGAAGCACAGCUAAAAUUUGAAUUUCAGAUAAACAAAUUUUUCUUAGAAUAAGUAUGUCCGCAUGCAACAUGUGGGACAUACUUAUGCUAAAACAUUAUUUGUGUUUCAUCUGAAAUUCAAAUUCAACUGGACAUCCUGUAUUUU

>ponAbe2-mir-1302-6_chr7:66504483-66504572(+)

AACAAAUAAUUUGGUAAUAUAUGUAUGGCCCACACAAUAUUUAGGACAACAAUAUUUGGGACAUACUUAUGCUAAAAAAGUAUUUGUUGA

>ponAbe2-mir-1302-7_chr8:150103359-150103431(-)

ACAACAUUUUUUUUAGUACAUGUAUGUCUGGUGCAACAAUUGGGACAUACUUAUGCUAAAAAAAUUAGUGUUC

>ponAbe2-mir-1302-8_chr9:93124282-93124409(-)

CCCAUUUAAACUUGAAUUUCAUAUAAACAGAGUAAUUUUCAGCAUUAGUGUAUCACAUGCAGUAUUUGGGACAUACUUAUGCUAAAAAAUUAGGUGGUGUUGAUCUGAAAUUCCAGUGUAGAUGGGCA

>proCap1-mir-1302-1_scaffold:5893_6075-6218(-)

CAUGACAUUCAGUUACAUCUGAAUUUCUGAACAGCAAUGAAUACAUUUUUGUGUAAGUAUGACCCAUGCAAUAUUUGGGACAUACUUAUGCUGAAAAUUUGUUCAUUGUGUAUCUGAAAUUCAAAUGUAACUGGGAGUCCUGCA

>pteVam1-mir-1302-4_scaffold:6820_23786-23937(-)

AAUAAAGUAGCACAGCUAAUUUUGAACUUCAGAUAAACAAUAAAUAUUUUUAAAGAAUAAGUAUGUCUCAUACAAUAUUUGAGACAUCUUUAUGCUAAAACAUUAUUUGUGUUUCAUCUAAAAUUCAACUUUAAUUGAACAUCCUGUAUUUU

>pteVam1-mir-1302-8_scaffold:8076_61659-61779(-)

CCCAGUUAAUCUUGAAUUUCAAAUAAGCAAUGAGUAAUUUUUAGCGUAAAUAUGUCCCAUGCAAUAUUCGAGACAUACUAAAAAUAUUAUGUCUUUUAUCCGAAAUUCAAAUUUAACUGGG

>rheMac2-mir-1302-2_chr11:10197-10333(+)

GGAUGCCCAGUUAGUUUGAAUUUUAGAUAAACAACGAAGAAUUUCUUAGCAUAAAUAUGUCCCAAGCUUAGUUUGGGACAUACUUAUGCUAAAAGUAUUAUUGGUGGUUUAUCUGAGAUUCAAAAUUAAGCAUUUUA

>rheMac2-mir-1302-4_chr12:71164776-71164925(-)

AAUGCAGAAGCACAGCUUAAAUUUGAAUUUCAGAUAAACAAAUUUUUCUUAGAAUAAGUAUGUCCCCAUGCAACAUUUGGGACAUACUUAUGCUAAAACAUUAUUUGUGUUUCAUCUGAAAUUCAAAUUCAACUGGACAUCCUGUAUUUU

>rheMac2-mir-1302-7_chr8:144301426-144301496(-)

ACAACAUUUUUUAGUACAUGUAUGUCUGGUGCAAUCAUUGGGACAUACUUAUGCUAAAAAAAUUAGUAUUC

>rheMac2-mir-1302-8_chr15:38854157-38854285(-)

CCCAUUUAAGUUUGAAUUUCAUAUAAACAGAGUAAUUUUCAGUAUUAGUGUAUCACGUGCAGCAUUUGGGACAUACUUAUGCUAAAAAAAUUAGGUGGUGUUGAUCUGAAAUUCCAGUGUAGAUGGGCA

>tarSyr1-mir-1302-7_scaffold_283147:3536-3608(-)

GCAAUUUUUUUCUUUAGUACAAGUAUGUCUGGUGCAAUAAUUAGGACAUACUUAUGCUAAAAAAUUAUCAUUC

>tarSyr1-mir-1302-8_scaffold_136008:2421-2543(-)

UCCAUUUAAACUUAAAUCUCACAUAAGCAAAAGAGCAAUUUUUAGUAUAAGUAUGUCCCAAGCAAUAUUUGGGACAUAUUUAUAUUAGAUGCUGUUGAUCUGAAAUUUCAAUUUAGCUGGGUA

>tupBel1-mir-1302-6_scaffold_120967.1:334544_60428-60504(-)

AAUAAAUAAUGGAGUAGUGUAUAUACAUGUCCUAUGCAAUACUGGGGACAUGUUUAUACUAAAAAAUGAUUUCUUGA

>tupBel1-mir-1302-8_scaffold_1835.1:209735_142412-142541(-)

UUCAGUUAAACCCGAGUUUCACGUAAGCAAAAAGCCAUUUUUAGUGUGAGUAUAUCCCAGUCAACAUGUGGCAUAUAGCUAUACUAAAAAAUUAGAUGUUGUCGAACUGAAAUUCCAGUUUAACUGAGCA

>turTru1-mir-1302-6_scaffold_112574:562939-563012(-)

AACAAAUAUUUGCAGUAUAAAUAUGUCCCAUGCAAUAUUAGGGACGUACUUGUAGCUAAAACAUUAUUUGUUGA

>vicPac1-mir-1302-4_scaffold_242:742657-742807(-)

AACACAGAAGCACAGCUAAUUUUGAAUUUCAGAUCGACAAUACGUAUUUUUUAGGAAUAAGUCUGUCCCAUGCAGUAUUUAGGACGUACUUAUGCCAAAACAUUAUUUGUGUUUCAUCUUAAAUUCAAAUUUAACUGGACAUCCUGUUUUU

>vicPac1-mir-1302-5_scaffold_4201:201332-201474(-)

UGCCCAGUUAAAUUUGAUUUUCAGAGAAAUAAAUCAUUUUCAGUACAAGUAUGUCCCAUGUAAUCUUUGGGACAUACUUAUACUAAAAAAGAAAUCUAUUUAUUGUUUAUUGGAAAUUUAAAUGUGACUGGGUGUCCUGUAUU

>vicPac1-mir-1302-6_scaffold_30:1405852-1405927(-)

AAUAAAUAUUUGCAGUAUAAGUACGUCCCAUGCAAUAUUGGGCACAUACCUGUACUCAAAAAAAAUUAUUUGUUGA

**References:**

1. Griffiths-Jones S, Grocock RJ, van Dongen S, Bateman A, Enright AJ: **miRBase: microRNA sequences, targets and gene nomenclature**. *Nucleic Acids Res* 2006, **34**(Database issue):D140-144.

2. Kent WJ, Sugnet CW, Furey TS, Roskin KM, Pringle TH, Zahler AM, Haussler D: **The human genome browser at UCSC**. *Genome Res* 2002, **12**(6):996-1006.

3. Kent WJ: **BLAT--the BLAST-like alignment tool**. *Genome Res* 2002, **12**(4):656-664.

4. Jiang P, Wu H, Wang W, Ma W, Sun X, Lu Z: **MiPred: classification of real and pseudo microRNA precursors using random forest prediction model with combined features**. *Nucleic Acids Res* 2007, **35**(Web Server issue):W339-W344.

5. Lee AS, Gutierrez-Arcelus M, Perry GH, Vallender EJ, Johnson WE, Miller GM, Korbel JO, Lee C: **Analysis of copy number variation in the rhesus macaque genome identifies candidate loci for evolutionary and human disease studies**. *Hum Mol Genet* 2008, **17**(8):1127-1136.

6. Yang MQ, Taylor J, Elnitski L: **Comparative analyses of bidirectional promoters in vertebrates**. *BMC Bioinformatics* 2008, **9 Suppl 6**:S9.

7. Toleno DM, Renaud G, Wolfsberg TG, Islam M, Wildman DE, Siegmund KD, Hacia JG: **Development and evaluation of new mask protocols for gene expression profiling in humans and chimpanzees**. *BMC Bioinformatics* 2009, **10**:77.

8. He X, Ling X, Sinha S: **Alignment and prediction of cis-regulatory modules based on a probabilistic model of evolution**. *PLoS Comput Biol* 2009, **5**(3):e1000299.

9. Walser JC, Ponger L, Furano AV: **CpG dinucleotides and the mutation rate of non-CpG DNA**. *Genome Res* 2008, **18**(9):1403-1414.
